# Factors Related to Change in Depression among North Korean Refugee Youths in South Korea

**DOI:** 10.3390/ijerph16234624

**Published:** 2019-11-21

**Authors:** Subin Park, Soo Yeon Kim, Eun-Sun Lee, Jin Yong Jun

**Affiliations:** 1 Department of Research Planning, Mental Health Research Institute, National Center for Mental Health, Seoul 04933, Korea; oss1793@daum.net (S.Y.K.); chachagrace918@gmail.com (E.-S.L.); 2 Department of Psychiatry, National Center for Mental Health, Seoul 04933, Korea; jjy826@korea.kr

**Keywords:** depression, North Korean Refugee Youths, protective factors, resilience theory

## Abstract

This study investigated change in depression and revealed factors related to change using one-year follow-up data. A sample of 108 North Korean Refugee Youths (NKRYs) aged 13 to 26 years (66 females) was recruited from two alternative schools for NKRYs in South Korea. Based on the Center for Epidemiologic Studies Depression Scale cut-off score of 16, respondents were grouped based on change in depression score after one year as stable low, alleviated, deteriorated, or prolonged. Multinomial logistic regression assessed the influence of baseline psychological scores (resilience, emotional regulation strategy, and self-esteem), and reported social support (psychological/practical) on the odds of group classification. With the stable low group as the reference category, those with alleviated depression at Time 2 had significantly higher odds of expressive suppression and tended to have lower self-esteem at Time 1. The deteriorated depression group was more likely than the stable low group to have lower resilience and cognitive appraisal scores. Those with prolonged high depression were more likely than the stable low group to have lower resilience, less practical social support, and lower self-esteem. Psychological interventions, particularly those focused on increasing self-esteem and resilience, could be helpful for NKRYs with potential risk of depression. In addition, practical support should be provided on an as-needed basis to prevent chronic depression among NKRYs.

## 1. Introduction

According to Ministry of Unification [1], in 2018, an estimated 32,476 North Korean refugees (NKRs) had settled in South Korea, about 40% of which were children and young adults aged 10 to 29. Scholarly interest in NKRs has increased along with their growing numbers during the past 10 years, but there is scant research on them, particularly North Korean Refugee Youths (NKRYs). NKRs are likely to have had traumatic experiences, such as compulsory confinement, arrests and detainments, witnessing people die of starvation, witnessing public executions or accidents, or being deported to North Korea [2]. NKRYs have reported a variety of traumatic experiences while they were in North Korea and on their way to South Korea [3]. NKRYs often feel a sense of guilt and loss about family members that were separated from them during their escape, and they have fears about others’ knowledge of them as refugees [3,4].

NKRYs often suffer from post-traumatic stress disorder (PTSD), depression, or anxiety because of these traumatic events [5]. In particular, depression, which is a psychological symptom of maladjustment, is a key indicator used to assess the mental health of immigrants and refugees [6], and it is one of the most common maladjustment problems of NKRs [7]. Because childhood depression might cause psychosocial and other problems in adulthood, it might be associated with a huge social cost [8]. Therefore, it is important to relieve the depression experienced by NKRYs and prevent their childhood problems from affecting their adult lives.

Adolescence is usually a vulnerable stage in the life course when physical, interpersonal, cognitive, environmental, and stress problems can negatively influence mental health [9]. NKRYs are particularly vulnerable to internal problems, such as anxiety and depression, and their depression is likely to increase over time after they arrive in South Korea [2,10]. However, resilience theory proposes that the developmental outcomes of adolescents who spent their childhoods in stressful environments, such as poverty or abuse, or those who experienced adverse events, are not necessarily negative [11,12,13,14]. Consequently, researchers also consider protective factors. Protective factors are expected to reduce the likelihood of dysfunction or illness after life experiences that might cause vulnerability and/or stress [15]. Adolescent developmental protective factors [16,17,18] are classified as personal characteristics and internal factors (e.g., self-esteem), positive responses to others, and cognitive factors; social supporters or adults who warmly care for and supervise youths; and adult support systems, social supports, adult role models, and service institutions that link youths to their communities. Therefore, clarifying the personal and environmental factors associated with change in depression is important to realizing effective interventions.

Many studies on NKRYs’ mental health have focused on psychopathology, such as suicidal ideation, depression, anxiety, and PTSD, and the associated risk factors. However, little research has been conducted on the protective factors, such as positive mental health, character strength, and social support. Adaptation to a new culture in South Korea places constant demands on every aspect of NKRYs’ lives, and, because their lives are dynamic, it is important to conduct longitudinal analyses to examine changes in mental health. Based on the previous studies about these youths and their mental health, this study examined changes in depression levels and the related protective factors and risk factors in a sample of NKRYs to obtain insight for effective interventions in accordance with the results of the current study.

## 2. Materials and Methods

### 2.1. Participants

A sample of NKRYs studying for their qualification examinations for high school or middle school graduation were recruited from two alternative schools for NKRYs. Since many NKRYs exhibit delayed educational processes due to their unique circumstances, these alternative schools provide more personalized education as well as additional cultural and psychological support to contribute to NKRYs’ integration in South Korea. Both schools volunteered to participate in the study as a part of a mental health screening program. We visited each school and requested their consent to participate in the study after explaining the questionnaire content and survey procedures. Of the 184 NKRYs who attended these schools, ten students declined participation, resulting in a total of 174 students who were enrolled in the study in 2017 and 2018 (baseline). Among them, 108 completed a one-year follow-up questionnaire in 2018 and 2019 (attrition rate: 37.9%) and were ultimately included in this study. The baseline questionnaire was a self-report instrument that asked about socio-demographic, personal, psychological, and social support characteristics as well as depression (T1). One year later, follow-up data were collected using the identical depression scales (T2). The Framework Act on Youth of South Korea defines youths as 9 to 24 years old. However, in North Korea, the term “youth” could encompass ages 6–13 years when receiving education, whereas it could also encompass ages 14–27 years when participating in political and social activities. Given this spectrum as defined by South and North Korea, we defined NKRYs as those aged 12–27 years. The study was reviewed and approved by the institutional review board of the National Center for Mental Health (No. 116271-2017-11).

### 2.2. Variables and Measurement

#### 2.2.1. Sociodemographic Characteristics

Gender, age, birthplace, parental education level, and type of household (i.e., with immediate family, other relatives, friends, alone, or in a facility) were the personal characteristics used in the study’s analysis.

#### 2.2.2. Potential Risk of Depression

The Center for Epidemiologic Studies Depression Scale (CES-D) was developed to measure depressive symptoms in the general population [19]. It has four positive items (e.g., “I felt that I was just as good as other people during the past week”) and sixteen negative items (e.g., “I felt that I could not shake off the blues even with help from my family or friends during the past week”). The response options are on a four-point scale where 0 = rarely or none of the time (less than one day per week), 1 = some or a little of the time (one or two days per week), 2 = occasionally or a moderate amount of time (three to four days per week), and 3 = most or all of the time (five to seven days per week). The four positive items were reverse-coded; and, then, the responses were summed. The total scores ranged from zero to 60, 16 through 25 points was classified as mild depression and more than 25 points was classified as clinically significant major depression [20]. This classification was based on the usual 16-point cut-off used in primary care [21]. For the analysis, depression levels were (1) low = zero to 15 and (2) high = 16 or higher.

#### 2.2.3. Psychological Characteristics

Three psychological characteristics (i.e., self-esteem, resilience, and cognitive style) were measured. The Brief Resilience Scale (BRS) measured resilience defined as the process of, or capacity for, successful adaptation after exposure to trauma or severe stress [22,23]. The scale has three positive items (e.g., “I tend to bounce back quickly after hard times”) and three negative items (e.g., “I have a hard time making it through stressful events”). The response options are on a five-point scale where 1 = strongly disagree through 5 = strongly agree. The negative items were reverse-coded, and the scores were then summed for a composite score, which ranged from six to 30, with higher scores indicating more resilience.

The Emotion Regulation Questionnaire (ERQ) measured cognitive reappraisal (e.g., “When I want to feel more positive emotions, I change the way I’m thinking about the situation”) and expressive suppression (e.g., “When I am feeling negative emotions, I make sure not to express them”), which are two emotional regulation strategies [24]. The response options were on a five-point scale where 1 = strongly disagree through 5 = strongly agree. Higher scores indicated that the use of more strategies to control emotions by changing their thoughts or suppressing emotions.

The Rosenberg Self-Esteem Scale was used to assess self-esteem [25]. It has six positive items (e.g., “On the whole, I am satisfied with myself”) and four negative items (e.g., “At times I think I am no good at all”). The response options were on a five-point scale where 1 = strongly disagree through 5 = strongly agree. The responses were summed after reverse-coding the negative items. Total scores ranged from 10 to 50, with higher scores indicating higher self-esteem.

#### 2.2.4. Social Support

Two items were used to measure social support as psychological or practical support received from others. Psychological support was measured by responses to the question: “How much psychological support do you currently receive from your family, relatives, friends, and others around you?” Practical support was measured by responses to the question: “How much practical support do you currently receive from your family, relatives, friends, and others around you?” Both items’ response options were on a 10-point scale where 1 = none at all through 10 = enough.

### 2.3. Statistical Analyses

First, descriptive statistics were generated on all the variables. We then compared the two groups (e.g., those with follow-ups vs. those without) to see if their baseline scores differed. A multinomial logistic regression analysis was performed to identify the variables significantly related to change in depression by comparing T2 to T1 data. Odds ratios (ORs) and 95% confidence intervals (CIs) were calculated using the group factor as the outcome variable, and sociodemographic, personal, and psychological variables were predictors.

The outcome variable was based on the CES-D cut-off score of 16. Depression change was categorized using the CES-D scores at T1 and T2: stable low (T1 low and T2 low), alleviated (T1 high and T2 low), deteriorated (T1 low and T2 high), and prolonged (T1 high and T2 high). All statistical analyses were performed using SPSS 20.0 (IBM, Armonk, NY, USA), and the statistical significance cut-off level was *p* < 0.05.

## 3. Results

The sample comprised 66 female and 42 male NKRYs aged 13 to 26 years old with a mean age of 17.84 (SD = 2.45 years). Table 1 presents the descriptive statistics. Twelve of the 66 respondents who scored high on depression symptoms at T1 had lower scores at T2 (alleviated). Fifty-four of the 66 respondents who scored high on depressive symptoms at T1 also had high depression scores at T2 (prolonged), and 20 of the 42 respondents with low depression scores at T1 had higher scores (>16) at T2 (deteriorated). There was no significant difference in socio-demographic variables (i.e., gender, age, birthplace, parental education level, and residence) in the four depression change groups.

When comparing baseline scores between the follow-up group and the group without follow-ups, there was a significant difference in mean age (20.35 ± 3.11 years in the group without follow-ups vs. 17.84 ± 2.45 years in the follow-up group, *p* < 0.001). There was no significant difference in gender (girls = 72.4% in the group without follow-ups vs. 61.1% in the follow-up group, *p* = 0.076). There were no significant differences in baseline scores on the CES-D, BRS, RSES, or ERQ (p > 0.05). There was no significant difference in psychological support (*p* = 0.126), but there was a significant difference in practical support (5.95 ± 2.83 in the group without follow-ups vs. 6.99 ± 2.81 in the follow-up group, t = 2.36, *p* = 0.02).

Table 2 presents the results of the multinomial logistic regression analysis of the predictors of change in depression. The alleviated depression group had significantly higher odds than the stable low depression group regarding expressive suppression and tended to have low self-esteem at T1. The deteriorated depression group was more likely than the stable low depression group to have low resilience and low cognitive reappraisal at T1. The respondents with prolonged depression were more likely than the stable low group to have low resilience, low self-esteem, and perceive low practical support at T1.

## 4. Discussion

This study investigated factors related to change in the potential risk of depression among NKRYs focusing on the progression, maintenance, and alleviation of depression. The NKRYs whose depression was alleviated between T1 and T2 had significantly higher T1 expressive suppression scores and tended to have lower T1 self-esteem scores than those in the stable low group. Low self-esteem and expressive suppression are two features of depression [24,26,27,28]. Therefore, the relatively low self-esteem and relatively high expressive suppression scores of the respondents in the alleviated depression group (compared to the stable low group) seem to reflect their high baseline depression scores rather than that these factors alleviated their depression. These results imply that programs to promote self-esteem and emotional expression might be effective interventions for depressed NKRYs.

The respondents in the deteriorated depression group had significantly lower resilience and lower cognitive reappraisal scores at T1 than those who were in the stable low group. Resilience is a protective factor previously associated with subjective health status and low depression and anxiety among NKRYs [29]. Resilience theory proposes that personal characteristics and internal factors (e.g., self-esteem and cognitive style) classified as protective factors reduce the likelihood of dysfunction among those with life experiences that could cause vulnerability or stress [15]. The ways that NKRYs perceive, interpret, and accept traumatic experiences or acculturation stress seems to be important. Considered together, low resilience and low cognitive reappraisal seemed to create a propensity to be vulnerable that increased the risk of depression among the respondents, which implies that depression prevention programs for NKRYs should focus on promoting resilience and enhancing healthy emotional regulation strategies.

This study’s prolonged depression group had significantly lower resilience, practical social support, and self-esteem scores at T1 than those in the stable low group. In addition to low resilience and self-esteem, which are historically considered central factors in depression [26,27,28], lack of practical social support seemed to have a significant role in depression maintenance. The relationships between depression and low self-esteem and social support could be bidirectional. Individuals with depression may be more likely to have low self-esteem [30] and feel socially isolated and as though they have a lack of social support [31]. These feelings of low self-esteem and insufficient social support, in turn, may be a risk factor for developing more severe depression [26,27,28,30,31,32]. The psychological and practical support received from family members, friends, and others were previously associated with NKRYs’ mental health [33,34]. These are protective factors in resilience theory because, through social support, individuals obtain knowledge and advice that helps them to adapt to their environment, improve their adaptability skills, and cope with change and stress [35]. In particular, practical support functions to adequately provide resources and services necessary for youths, thereby improving mental health by reducing stress and helping with environmental adaptation [33,34,35]. However, a previous study indicated that the average number per NKRYs of individuals providing practical, informational, and emotional assistance was 2.1 per person, and about 12% of the youth had no social supporters [33]. Those who lack social support are expected to be relatively vulnerable to mental health problems caused by stress.

To the best our knowledge, this is the first longitudinal study on change in the potential risk of depression among NKRYs. The findings provide insights into the variation in depression among NKRYs. First, relatively low resilience at T1 related to increased depression at T2, suggesting that resilience is a risk factor for deteriorated depression. Second, because self-esteem was significantly lower in the T1 high depression group, low self-esteem seems to be important to the initial state of depression. Third, low practical social support seems to be a factor that contributes to maintaining a high level of depression. These results will help develop programs to improve mental health among NKRYs and inform experts on depression. Further studies are needed on larger and representative samples, and the protective effects of family characteristics should be analyzed to verify our results.

Despite this study’s important findings, it has several limitations. First, the sample was small, with only 12 to 50 people per depression change group, which might have influenced the external validity of the results. Second, social support was measured with two single items. Further research is needed that uses a validated scale to distinguish among social supports received from family members, friends, and others. Third, we did not collect any information on treatment for depression, which could have affected changes in depression during the follow-up period. Finally, this study revealed factors associated with change in depression among NKRYs, but it did not explore the ways that these factors might relate to alleviation, deterioration, or maintenance of depression. Longitudinal studies of longer duration, with more follow-ups, and that use statistical analyses, such as temporal mediation or moderation, would help to reveal the relevant mechanisms.

## 5. Conclusions

This study examined the factors associated with change in the potential risk of depression and those that affect the progression, maintenance, and alleviation of depression among NKRYs. Twelve of the 66 respondents who scored high on depression symptoms at T1 had lower scores at T2 (11.1%; alleviated). A total of 54 participants scored high on depressive symptoms at T1/2 (50.0%; prolonged), and 20 respondents with low depression scores at T1 had higher scores (>16) at T2 (18.5%; deteriorated). The alleviated depression group had significantly higher odds than the stable low depression group regarding expressive suppression and tended to have low self-esteem at T1. The deteriorated depression group was more likely than the stable low depression group to have low resilience and low cognitive reappraisal at T1. The respondents with prolonged depression were more likely than the stable low group to have low resilience, low self-esteem, and perceive low practical support at T1. The results suggest that depression prevention programs and policies for NKRYs should focus on promoting self-esteem, resilience, healthy emotional regulation strategies, and concentrating on providing social supports.

## Figures and Tables

**Table 1:** Characteristics of participants (*n* = 108).

Characteristics	*n* (%) or M (SD)
Gender	
Male	42 (38.9)
Female	66 (61.1)
Birthplace	
North Korea	47 (43.5)
China	61 (56.5)
Paternal education level	
High school degree or less	59 (54.6)
College degree or more	19 (17.6)
Maternal education level	
High school degree or less	49 (45.4)
College degree or more	29 (26.9)
Residence	
Immediate family	51 (47.2)
Other relatives, friends, alone, or in a facility	57 (52.8)
Depression change	
Stable low	22 (20.40)
Alleviated	12 (11.11)
Deteriorated	20 (18.52)
Prolonged	54 (50.00)
Psychological characteristics	
Resilience	33.83 (6.47)
Self-esteem	18.83 (4.92)
Cognitive reappraisal	19.65 (3.35)
Expressive suppression	11.76 (2.53)
Social support	
Psychological	6.59 (2.81)
Practical	6.59 (2.81)

**Table 2:** The effects of the sociodemographic and psychological factors on change in depression; adjusted odds ratios (AOR) and 95% Confidence Intervals (CI).

Variable	Depression Change (Ref: Stable Low Depression, *n* = 20)								
	Alleviated Depression (*n* = 12)			Deteriorated Depression (*n* = 20)			Prolonged Depression (*n* = 54)		
	AOR	95% CI		AOR	95% CI		AOR	95% CI	
Gender	0.60	0.10	3.69	1.30	0.28	6.15	0.94	0.23	3.89
Age	0.91	0.56	1.45	0.81	0.57	1.15	0.86	0.62	1.17
Psychological characteristics									
Resilience	0.88	0.69	1.12	0.80 *	0.66	0.97	0.77 **	0.64	0.93
Self-esteem	0.82 *	0.68	0.99	1.08	0.92	1.25	0.85 *	0.74	0.99
Cognitive reappraisal	0.76	0.55	1.03	0.75 *	0.58	0.96	0.91	0.70	1.18
Expressive suppression	1.78 **	1.15	2.74	1.20	0.86	1.67	1.26	0.94	1.69
Social support									
Psychological	1.04	0.71	1.52	0.88	0.66	1.17	0.89	0.67	1.17
Practical	0.85	0.53	1.36	0.82	0.58	1.16	0.79 *	0.49	0.98

* *p* < 0.05, ** *p* < 0.01; *Abbreviations:* The stable low depression group (*n* = 20) was treated as the reference; AOR, adjusted odds ratio; adjusting for all variables in Table 2; CI, confidence interval.
